# Advancements and Challenges in Computer-Assisted Navigation for Cervical Spine Surgery: A Comprehensive Review of Perioperative Integration, Complications, and Emerging Technologies

**DOI:** 10.1177/21925682251329340

**Published:** 2025-04-04

**Authors:** Hania Shahzad, Aziz Saade, Shannon Tse, Samuel Simister, Anthony Viola, Sathish Muthu, Hardeep Singh, Luca Ambrosio, Javad Tavakoli, Sven Yves Vetter, Philip Louie, Samuel Cho, Sangwook Tim Yoon, Amit Jain, Hai Le, AO Spine Knowledge Forum Degenerative, AOSKFDegenerative@aofoundation.org

**Affiliations:** 1 70083 UC Davis Health, Sacramento, CA, USA; 2 Department of Orthopaedics, Orthopaedic Research Group, Coimbatore, India; 3 612500 Department of Spine Surgery, Orthopaedic Research Group, Coimbatore, India; 4 Central Research Laboratory, Meenakshi Medical College Hospital and Research Institute, Meenakshi Academy of Higher Education and Research, Chennai, India; 5 21654 University of Connecticut Health Center, Farmington, CT, USA; 6 Research Unit of Orthopaedic and Trauma Surgery, Department of Orthopaedic and Trauma Surgery, Università Campus Bio-Medico di Roma, Rome, Italy; 7 Operative Research Unit of Orthopaedic and Trauma Surgery, Fondazione Policlinico Universitario Campus Bio-Medico, Rome, Italy; 8 5376 School of Engineering, RMIT University, Melbourne, VIC, Australia; 9 72068 BG Unfallklinik Ludwigshafen, Ludwigshafen, Germany; 10 Virginia Mason Medical Center, Seattle*,* WA, USA; 11 Department of Orthopaedic Surgery, 5925 Icahn School of Medicine at Mount Sinai, New York, NY, USA; 12 Emory University, Atlanta, GA, USA; 13 1501 Johns Hopkins Medicine, Baltimore, MD, USA

**Keywords:** computer-assisted navigation, cervical spine surgery, complications, augmented reality, robotics, surgical accuracy

## Abstract

**Study Design:**

A narrative review of the current literature on the application of Computer-Assisted Navigation (CAN) in cervical spine surgeries.

**Objective:**

To analyze the perioperative integration, types of CAN systems, technical considerations, and clinical applications of CAN in cervical spine surgeries, as well as to assess the associated complications and potential strategies to minimize these risks.

**Methods:**

A comprehensive review of published studies between 2015 and 2024 was conducted to evaluate the usage, benefits, and challenges of CAN in cervical spine surgeries. The review covered perioperative integration, system types, complications, and emerging technologies, including augmented reality (AR) and robotics.

**Results:**

The use of CAN in cervical spine surgeries provides improved accuracy in screw placement and reduced neurovascular complications. However, the review identified several limitations, such as a steep learning curve, cost considerations, and potential inaccuracies related to cervical spine mobility.

**Conclusions:**

CAN offers significant benefits in cervical spine surgeries, including enhanced precision and reduced complications. Despite the current limitations, advancements in AR and robotics hold promise for improving the safety and effectiveness of CAN in cervical procedures. The future focus should be on overcoming the existing challenges to increase the adoption of CAN in cervical spine surgeries.

## Introduction

Computer-assisted navigation (CAN) use in spine surgery has grown significantly, with reported cervical cases increasing approximately ten times from 2014 to 2018, with a decrease in elective procedures and an increase in nonelective ones for cervical cases.^1^ CAN has increased surgeons’ confidence in using this technology for complex, atypical, non-elective cases. However, its adoption and indications for cervical cases still need to be more widespread and evidence-based than thoracolumbar procedures. This is potentially due to the complex anatomy of the cervical spine, where smaller pedicles and the proximity of screws to vital neurovascular structures such as the vertebral artery, esophagus, spinal cord, and nerve roots present inherent surgical challenges.^2^ Additionally, there is a greater degree of motion while instrumenting the cervical spine, compared to the thoracolumbar spine leading to greater inaccuracies, with increased risk of catastrophic complications specific to cervical spine especially in the mid-cervical spine. This necessitates a higher degree of care to avoid moving the cervical spine during the placement of surgical instruments and reference arrays to prevent iatrogenic damage to these critical structures.^3^

Literature regarding the use of intraoperative navigation in cervical spine surgeries warrants a comprehensive review to objectively represent the challenges and technical considerations of implementing this technology. As technology advances and surgeons become more adept with navigation systems, their application in cervical spine surgeries continues to grow. This manuscript aims to review common complications that arise with the use of navigation in cervical spine surgery and to provide best practice recommendations to avoid these complications.

## Perioperative integration of CAN systems

CAN integration in cervical spine surgery involves preoperative planning and intraoperative navigation (Table 1). Preoperative imaging modalities such as plain radiographs, ultrasound, and CT scans are crucial, with CT being the cornerstone for detailed osseous anatomy. Some systems (e.g., Brainlab) also allow for MRI-CT fusion. They are crucial for planning surgical approaches, instrumentation, and potentially generating patient-specific implants. However, limitations include radiation exposure and the need for meticulous registration due to posture changes between preoperative supine and intraoperative prone positions. Intraoperatively, CAN systems can be imageless (using optical markers and referencing preoperative CT) or image-based utilizing 3D fluoroscopy or intraoperative CT-based navigation for real-time spatial referencing. Newer technologies like augmented reality (AR) and robotics enhance intraoperative CAN capabilities by providing real-time, three-dimensional visualization of critical structures potentially reducing neurovascular complications, and increasing precision.

**Table 1.** Utility of Computer-Assisted Navigation Systems in the Surgical Cycle of Cervical Spine Surgery.

Pre-operative strategic planning
• Strategic planning using radiographs, ultrasound, MRI, and CT scans
• Challenges include increased pre-operative time for imaging, planning, data processing, and system setup.
• Wide exposure during surgery, meticulous registration, and radiation exposure considerations.
• Shift to intraoperative CAN is necessary due to data dependency between preoperative and intraoperative positions.

Intra-operative utility	
Image-less	Image-based
• Use optical markers for navigation,	• Use markers in the operative field
• Use preoperative CT images as references	• Use intraoperative imaging for navigation.
• E.g., *AR & robotics*	• E.g., fluoroscopy and intraoperative CT scanners
Active-navigation	Passive-navigation
• Utilizes supplementary devices to establish pre-determined anatomical landmarks.	• Assists surgeons via image guidance
	• Allows variability in procedural methods
• E.g., AR offers real-time visualization of critical structures based on preoperative CT imaging.	• E.g., Robotics reduce neurovascular complications and operating times.

Navigation can also be classified as active or passive. Active navigation utilizes supplementary devices like drill guides to establish pre-determined screw trajectories. Passive navigation assists the surgeon via image guidance, allowing variability in approach, whereas active navigation uses devices like customized jigs or robotic assistance for predetermined anatomical fixation, reducing neurovascular complications. Passive navigation offers advantages like reduced wait times and allows manual override in certain scenarios which is not possible in active systems but has a steeper learning curve.

## Types of CAN Systems Available in the Market

CAN systems available in the market can be grouped into the type of radiological imaging that they use e.g., CT-based navigation (e.g., Airo Mobile Intraoperative CT-based platform), integrated platforms with combined modalities (e.g., Medtronic’s StealthStation with O-arm), fluoroscopy-based navigation (e.g., Ziehm Vision FD Vario 3-D system), optical navigation (e.g., Stryker’s SpineMask Tracker) and robotic-assisted navigation (ExcelsiusGPS by Globus Medical and the Cirq Robotic Alignment Module by Brainlab).

**Figure 1.**
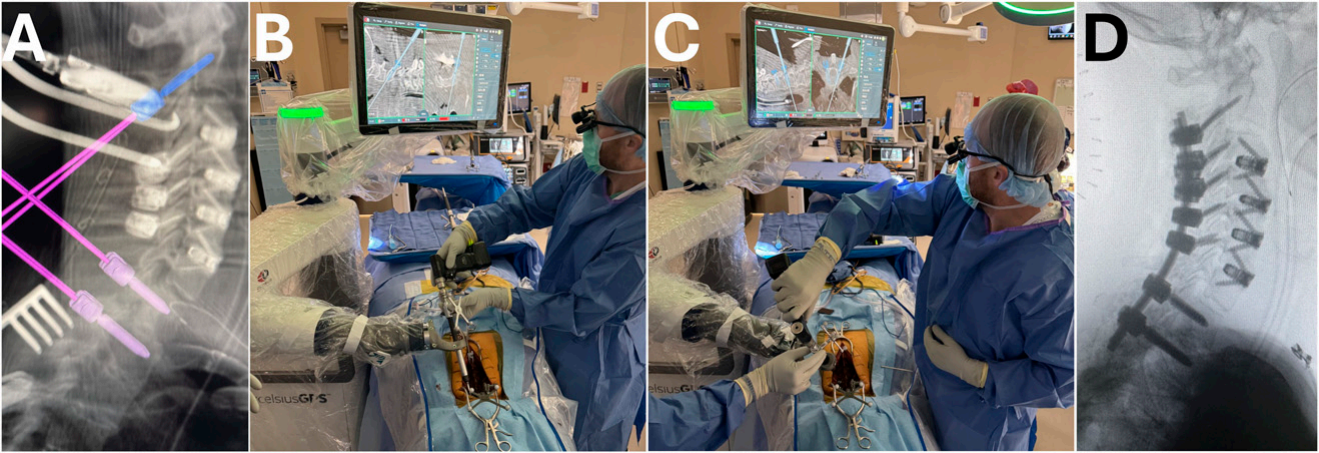
Robotic navigation for posterior cervical fusion. (A) Planned trajectories of C2, T1 and T2 pedicle screws using robotic navigation platform. Intraoperative photos of C2 pedicle screw (B) and T1 pedicle screw (C) placement using the robotic arm. Intraoperative lateral fluoroscopic imaging of the cervical spine status post C2-T2 posterior instrumented fusion.

The Airo Mobile system, a CT-based navigation platform, pioneered mobile intraoperative navigation using a large circular scanner attached to the operating table. This system provides real-time 3D mapping for instrumentation guidance, with instruments equipped with 3 reference points recognized by the system’s scanning stereotactic camera. Both Medtronic’s StealthStation with O-arm and the Ziehm Vision FD Vario 3-D system use integrated platforms with combined modalities for 360-degree imaging technology. The O-arm, part of electromagnetic navigation, is a mobile scanner with a 90-degree open configuration, allowing easy mobilization around the patient, while the Ziehm Vision FD Vario 3-D system is fluoroscopy-based and captures images through a 190-degree rotation around the patient. The Stryker SpineMask Tracker, an optical navigation system, offers an alternative referencing method by applying reference trackers directly onto the patient’s skin, making it suitable for minimally invasive surgery. However, caution is required to maintain accuracy, especially in cases of deep retraction or undue skin tension. As for robotic-assisted navigation, 2 systems have FDA clearance for use in cervical spine surgeries: the ExcelsiusGPS by Globus Medical and the Cirq Robotic Alignment Module by Brainlab. These systems require simultaneous intraoperative fluoroscopic confirmation or imaging workflow for real-time visualization.^4^ An intraoperative 3D scan is generally sufficient for planning and placing implants with both robotic systems (Figure 1).

## Technical or Surgical Considerations

Despite documented benefits in spinal instrumentation, including decreased pedicle violation rates, reduced operative times, and fewer revisions, evidence for CAN applications in the cervical spine remains limited.^5,6^ Screw placement in the cervical spine is particularly challenging due to the complex anatomy and narrow margins for error compared to the thoracolumbar spine necessitating careful technical considerations. Studies have reported an accuracy of 89-91.3% accuracy of screw placement without navigation in the cervical spine and a screw deviation rate of 2.5- 29.1%,^7,8^ where risks for damaging contiguous neurovascular structures can not be eliminated (Table 2).

**Table 2.** Surgical Considerations and Potential Applications of Computer-Assisted Navigation in Cervical Spine Surgery.

Segment	Technical Considerations	Utility of CAN
Atlantoaxial (C1-C2)	• Complex anatomy	• Optimizes screw placement using C1 lateral mass & C2 pedicle screws
	• Risk of injuring spinal cord, vertebral artery, C2 nerve root	• Applicable in minimally invasive procedures
	• Anomalous vertebral artery or high-riding transverse foramen	
C1 lateral mass	• Risk of injuring spinal cord (medially), vertebral artery (laterally), C2 nerve root (caudally), posterior arch of atlas (cranially)	• Minimizes risk of injuring critical structures
	• Potential damage to venous plexus and internal carotid artery (within 4.8 ± 2.7 mm, minimum 1.1 mm)	• Assists in planning insertion point & screw length
C2 pedicle screw	• High-riding vertebral artery	• Overcomes challenges of pedicle axis view with CT-based navigation
	• Difficulty in obtaining adequate pedicle axis view due to patient positioning and equipment	• Improves accuracy of screw placement
C2 translaminar screw	• Asymmetric screw placement to avoid midline intersection • Risk of screw collision & spinal canal breaching	• Defines precise insertion point, screw length, and trajectory with 3D navigation
		• Prevents screw collisions
C3-C6 pedicle screw	• Smaller pedicle size and narrow space between vertebral artery and pedicle	• Plans safe and reliable screw placement path
	• Limited safety for pedicle screw placement	
C7 pedicle screw	• Less concern for vertebral artery injury, but 0.3% risk remains	• Enhances anatomical visualization
	• Risk of C8 nerve root injury	• Lowers rates of screw misplacement
	• Spinous process clamps may hinder LMS placement	• Facilitates PS and LMS placement
Anterior cervical (ACDF/Corpectomies)	• Need to avoid vertebral artery during decompression or bone removal	• Improves visualization during decompression & bone removal
	• Critical for foraminal stenosis and tumor cases	• Provides clear delineation of surgical borders

Due to the proximity of the vertebral artery, nerve root, and spinal cord with its widest transverse diameter in the cervical region,^9^ precise instrumentation is essential to prevent loss of construct stability or devastating neurovascular complications. Such complications are mainly caused by screw malpositioning. Intraoperative CAN may aid in establishing a safe corridor, by accurately planning screw trajectory and length.

Atlantoaxial instability surgery is notoriously risky due to the complex anatomy of the upper cervical spine.^10^ While posterior transarticular screws offer strong biomechanical stability, up to 23% of patients may have an anomalous vertebral artery course or high-riding transverse foramen at C2.^11-13^ CAN optimize screw placement in a previously described technique using C1 lateral mass and C2 pedicle screws.^14,15^ This approach offers comparable stability to transarticular screws and is applicable in minimally invasive percutaneous procedures.^16^

Due to the complex anatomy surrounding the C1 lateral mass, proper screw placement can be achieved with the use of CAN, which minimizes the risk of injuring the spinal cord medially, the vertebral artery laterally, the C2 nerve root caudally, and the posterior arch of the atlas with its overlying vertebral artery cranially.^14,17^ Furthermore, C1 lateral mass screw placement can also damage the venous plexus surrounding the starting point and the internal carotid artery located anteriorly (within 4.8 ± 2.7 mm and a minimum of 1.1 mm). Intraoperative CAN, especially if equipped with vascular delineations^18^ offers a valuable solution by assisting in planning the optimal insertion point and screw length, thereby reducing the risk of such complications.^4^

C2 pedicle screw fixation can be challenging due to a potentially high-riding vertebral artery positioned further cranially. This necessitates careful planning of screw length and trajectory. Obtaining an adequate pedicle axis view using AP/lateral fluoroscopy can be difficult because of the patient’s prone reverse Trendelenburg position, the Mayfield frame, and the lines or cables surrounding the patient. However, CT-based CAN has shown promise in overcoming these limitations to improve the accuracy of screw placement.^4^ C2 translaminar screw instrumentation, relying on the asymmetric placement of bilateral screws, offers an alternative to C2 pedicle screw insertion for patients with C2 bone or vascular anatomic variations.^19^ These screws require different starting points to prevent them from intersecting when crossing the midline. Consequently, 3D-CAN helps avoid screw collisions and spinal canal breaches by precisely defining the insertion point, screw length, and trajectory.^19^

While lateral mass screw fixation is more commonly used in the mid-cervical spine region (C3-C6), pedicle screw instrumentation is considered for patients with osteoporosis which provides superior biomechanical stability and lowers the risk of screw pullout compared to lateral mass screw.^20,21^ Additionally, pedicle screws can be used in post-laminectomy patients where anatomical landmarks are altered, making lateral mass screw placement unfeasible.^4^ The mid-cervical spine vertebrae have smaller pedicles, and the narrow space between the vertebral artery and the pedicle makes freehand pedicle screw placement challenging. In such scenarios, CAN is beneficial in planning safe and reliable pedicle screw trajectories for instrumentation.

At C7, surgeons are more likely to use pedicle screw instrumentation and are less concerned about injuring the vertebral artery, unlike other cervical levels. Still, caution should be applied to avoid devastating complications for 0.3% of patients with a vertebral artery entering the foramen transverse at C7.^22^ Additionally, careful consideration should be given regarding the vertical trajectory of the screw and its potential breaching risk to the exiting C8 nerve root which is essential for normal hand function^23^ Spinous process clamps, used as a reference array, are feasible at this level as it does not hinder PS placement. This is not the case for lateral mass screw placement as the screw is inserted from the contralateral side.^4^ In cases of both pedicle screw and lateral mass screw placement, CAN has the potential to enhance anatomical visualization leading to lower rates of screw malposition.^24^

In anterior cervical spine surgery (e.g., anterior cervical discectomy and fusion, cervical disc replacement, and cervical corpectomy), CAN improves visualization during decompression or bone removal. This is crucial for avoiding the vertebral artery, which runs close to the exiting nerves. CAN benefit both foraminal stenosis and tumor cases by providing clear delineation of surgical borders, especially during soft tissue or bone resections.^25^

### Clinical Applications

Achieving accuracy in the cervical spine is particularly challenging. Traditional fluoroscopy techniques have limitations, and even with advancements like O-arm navigation, questions remain regarding their specific benefits for cervical screw placement. While intraoperative CT-based navigation offers a significant improvement over freehand techniques by providing real-time guidance and improving screw placement accuracy,^26,27^ the persistent screw deviations of 2-4 mm remain a concern. This is likely due to the high flexibility of the cervical spine. Intraoperative CT captures a static image, unable to account for dynamic changes in spinal alignment during surgery. Minor movements, particularly impactful in the narrow cervical space, can compromise screw placement.^28^ Surgeons must remain vigilant for potential alignment shifts during screw insertion, similar to the freehand technique. Regularly confirming screw position by comparing navigation images with real-time palpation of the lamina or spinous process is crucial. Additionally, acquiring a post-placement CT scan in cervical spine surgery can further verify screw positioning.

Limitations due to the static nature of intraoperative 2D CT images give rise to intraoperative C-arm and O-arm navigation. A study comparing O-arm and C-arm navigation in cervical spine surgery included 448 screws placed with the O-arm and 670 with the C-arm.^29^ While the overall malposition rate was lower with O-arm imaging (0.93% vs 8.97%), a significant proportion of screws remained malpositioned in both groups, particularly in the cervical spine (18.75% vs 29.31%). This warrants further improvement in the reliability of these 3D intraoperative navigation systems for cervical spine surgeries.

**Figure 2.**
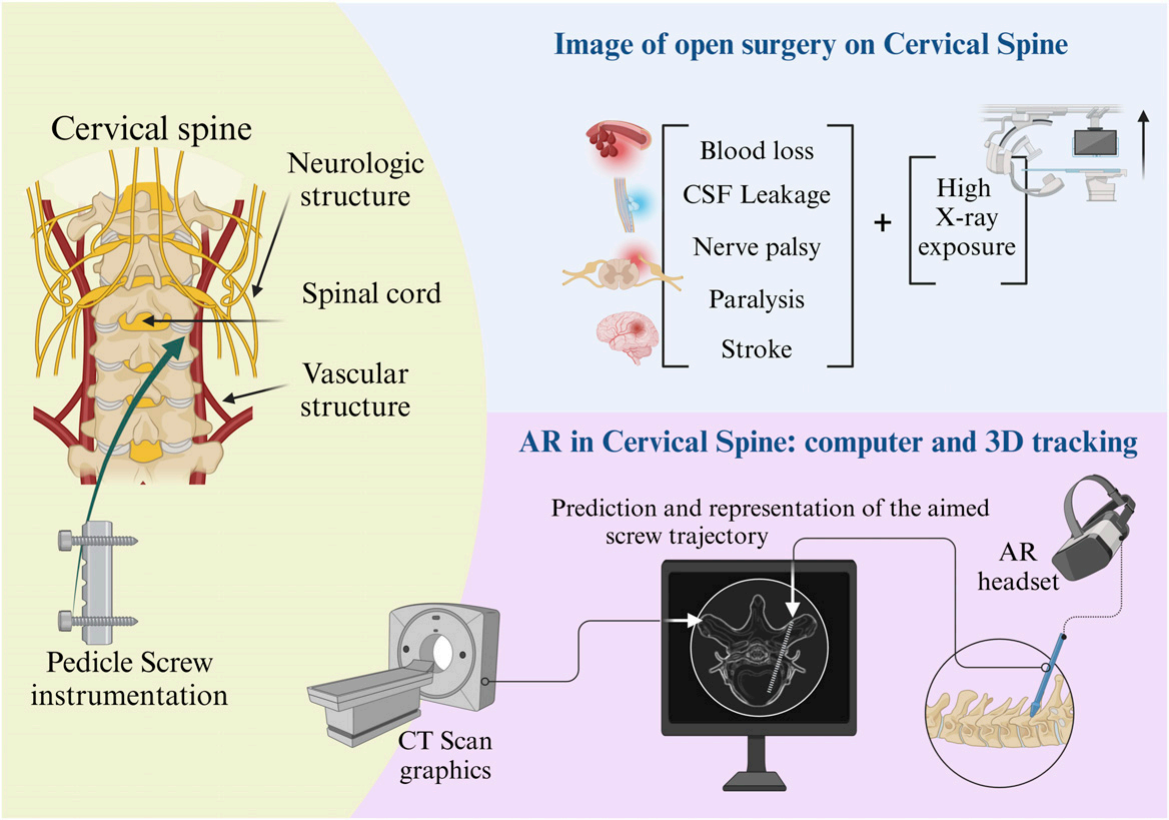
Visual comparison of traditional open surgery vs. AR-assisted techniques in cervical spine procedures: improving accuracy and reducing complications.

A recent study explored the use of O-arm intraoperative imaging to create a real-time AR navigation system for minimally invasive cervical spine surgery.^30^ This novel approach merged navigation data with a microscope, allowing surgeons to visualize critical structures not directly visible during surgery. The study reports successful decompression of spinal ossification and improvement in neurological symptoms for all patients. Importantly, no major complications were observed, thereby supporting the early feasibility and safety of AR-based navigation for cervical spine surgery (Figure 2).

### Augmented Reality

**Figure 3.**
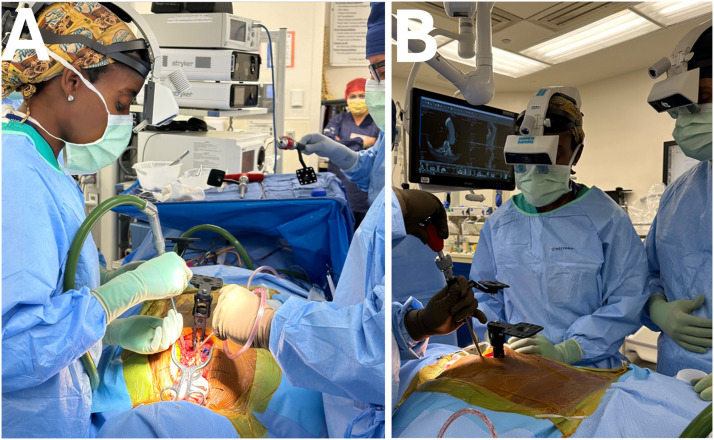
Augmented reality surgical navigation of thoracic spine. With AR navigation, the operating surgeon looks directly at the patient and visualizes the patient’s anatomy in 3D through a wearable transparent headset (A). The trainees or assistants also participate and visualize the surgery through similar headsets. (B). FDA-approved spine head-mounted display AR navigation systems.

The use of AR navigation in the cervical spine requires particular considerations and technical pearls differing from those of traditional navigation, robotics, or freehand screw placement confirmed with fluoroscopy. AR involves computer software correlating preoperative or intraoperative CT scans or 3D X-rays with the patient’s real anatomy in the operating room.^31^ In essence, the image created allows for an overlay of virtual information onto the surgeon’s view of the surgical field via an AR head-mounted display (AR-HMD) in the form of a headset, visor, or glasses. By superimposing the 3D reconstruction onto the surgical field, AR limits attention shift and enhances the surgeon’s depth perception allowing for a better understanding of the spatial relationships between various anatomical structures.^32^ This overlay of information in the surgeon’s line of sight in turn allows for precise localization of the spinal structures and critical landmarks and has shown increased accuracy of pedicle screw placement when compared to freehand, robotic, and other navigation systems (Figure 3A and B).^33-35^ The use of AR has also shown improved patient outcomes and reduced rates of reoperation after spine surgery when compared to traditional methods.^30^ Additionally, traditional methods for spine surgery instrumentation most often include significant amounts of radiation to the surgeon and operating room staff. AR navigation reduces radiation exposure to the surgeon and operating room staff as they can step outside of the room or behind a lead door during intraoperative CT imaging.^36^ Lastly, AR can serve as an excellent teaching tool for the surgeon as residents, fellows, and other medical providers who can view the imaging via the headset or viewing screen to learn the appropriate anatomical landmarks and screw trajectories which can lead to a reduction in the learning curve as seen when surgeons adopt new techniques.^34,35^

The advantages of AR in the cervical spine stem from the ability of AR to provide real-time visualization of anatomical structures and from the enhanced depth perception provided by AR when compared to other modalities. The cervical spine has several key vascular and neurologic structures that can lead to catastrophic effects if violated during pedicle screw instrumentation including paralysis, stroke, and even death. Real-time visualization with AR has led to similar results as compared to traditional techniques regarding intraoperative blood loss, CSF leakage, as well as postoperative nerve palsy and cervical alignment after anterior cervical discectomy and fusion or anterior cervical corpectomy and fusion.^30^ More so, this study showed improved patient outcomes as evidenced by decreased reoperation rates attributed to the ability to better assess and visualize anatomical structures and variants during these anterior cervical procedures.^30^ Other case series and case reports have reported successful postoperative outcomes and resolution of pain with the use of AR for posterior instrumentation of the cervical spine.^37,38^ Furthermore, studies have shown faster posterior cervical instrumentation with AR guidance, leading to increased operating room efficiency and potentially decreased operative time.^37^ Various kinds of AR set-ups are available but all these systems all have the advantage of being easy to use and having rapid registration (within 5 minutes). Also, studies show that they all demonstrate accuracy that rivals or is superior to freehand screw placement with the advantage of decreased radiation to the surgical staff.^39-41^

However, to date, there are very few studies on the use of AR in spine surgery and most of the literature demonstrates use in the thoracic and lumbar spine, not the cervical spine. Given that the lumbar and thoracic spine have larger and less delicate bone anatomy, it is difficult to prove that the documented accuracy of these systems correlates with their use in the cervical spine. Additionally, the limitations associated with this technology, particularly for new users, are expected to mirror those observed in thoracolumbar applications. These include cumbersome head-mounted displays, shifts in attention away from the operative field when using monitor-based systems, and extended exposure to virtual environments, which may result in physical and visual fatigue. However, these challenges may diminish as more technologically advanced equipment becomes available. Future studies will undoubtedly focus on the use of AR with these systems in the cervical spine to determine safety and accuracy (Table 3).

**Table 3.** FDA-Approved Spine Head-Mounted Display AR Navigation Systems.

Navigation System	Headset	Advantages	Disadvantages
Augmetics xvision (Arlington Heights, Illinois)	Augmetics xvision (Arlington Heights, Illinois)	Optical tracking cameras - real-time instrument tracking	Headset not commercially available
		Transparent near-eye display that projects an image onto the surgeon's retina	
		Vendor/implant neutral - reference markers easily attach to any instrument	Wireless headset but with battery cable - can tether on the gown or lead vest
		Navigation system/headset designed to link for ease of use	
		Rapid registration	Intraoperative CT required
		Headlight	
VisAR (Novarad, Provo, Utah)	HoloLens (Microsoft, Redmond, WA)	Commercially available headset	Short battery life (2-3 hours)
		Rapid registration	
		Voice-controlled to select images	Printing of QR codes with planned screw trajectories adds steps/time
		Hand tracking	
		Tether-less visor	Large visor frames
		Wifi, Bluetooth	
		Headlight	
SyncAR Spine (Surgical Theater, Cleveland, Ohio)	Magic Leap (Plantation, Florida)	Commercially available headset	Reference trackers are not easily attached to different vendor instruments/systems
		Real-time instrument tracking	
		Allows for inspection of the cross-sectional anatomy of the pedicle as instruments advance	
		Rapid registration	

### Technical Pearls

**Figure 4.**
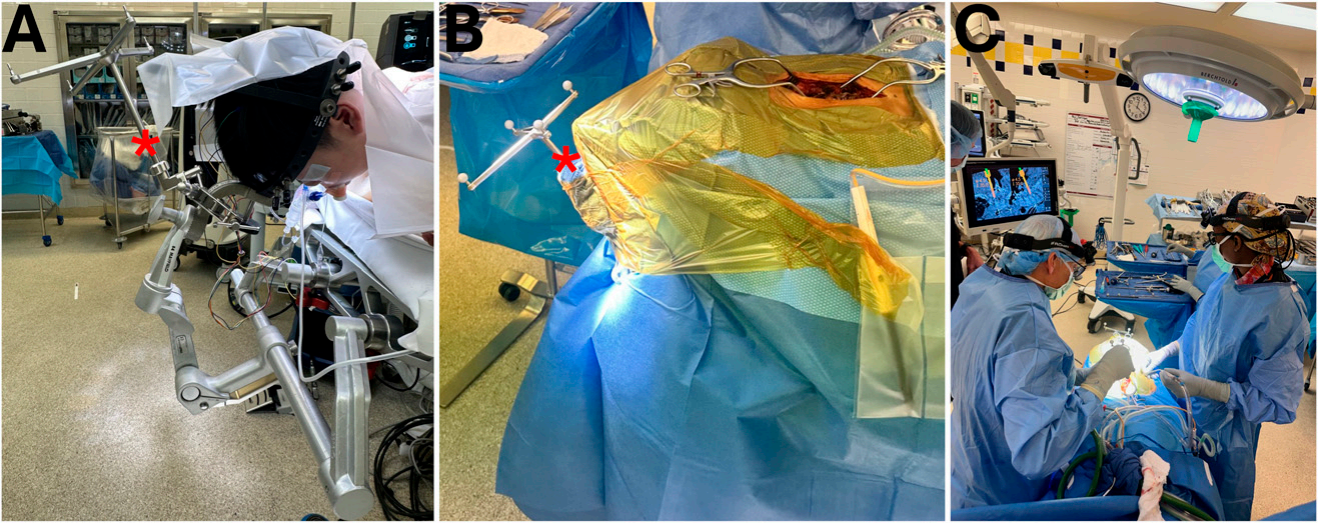
CT-guided navigation in a child with complex congenital cervical deformity. The reference array (*) is attached to the Mayfield cranial stabilization system (A), and sterile prepping and draping is performed around the array (B). The head of bed is turned 180 degrees away from anesthesia to facilitate intraoperative CT imaging and navigation. Intraoperative photo of thoracic pedicle screw (C) placement using CT navigation.

Incorporating CAN into the current state of cervical procedures requires several modifications. Detailed preoperative planning with CAN imaging allows for assessing complex anatomies, such as a high-riding vertebral artery, using sagittal, coronal, and axial views for accurate trajectory planning. Surgical exposure should be performed prior to registration to avoid dislodging the optical array. During surgery, Mayfield tongs can be used instead of Gardner-Wells tongs for a stable, non-rotational reference, with a rigid arm and reflective optical spheres attached through the surgical drape for direct line-of-sight visualization from the navigation camera placed at the foot of the bed. In some settings, the reference frame for navigation in cervical spine surgery may also be attached at the Mayfield tong instead of the spinous process (Figure 4). CAN-guided high-speed burrs can create precise pilot holes, and drill guides, taps, and screw inserters maintain accurate trajectories and depth control, minimizing the need for additional fluoroscopic imaging and reducing radiation exposure. When planning challenging trajectories with a robotic arm, separate incisions should be made to avoid soft-tissue pressure on the robotic arm. For subaxial cervical lateral mass screws, securing the reference array on a table-mounted or Mayfield-based holder is recommended due to prohibitive spinous process arrays. CAN is particularly beneficial for surgeries involving safe decortication and visualizing the C1 lateral mass starting point. Screw length and size are determined using intraoperative planning software, with navigated drill guides ensuring precise lateral mass drilling. Post-instrumentation scans should confirm all screw placements to verify accurate implant placement.^4^

### Barriers to Adoption and Future Directions for Navigation Technology in Spine Surgery

Pragmatic barriers hinder the widespread adoption of navigation technology in spine surgery. High implementation and production costs, limited evidence demonstrating its superiority, and intrinsic technological limitations contribute to its restricted use. Additionally, affordability remains a significant challenge, as many hospitals, particularly in low- and middle-income countries (LMICs), cannot afford these systems. The availability of this technology often favors academic centers over non-academic ones, raising concerns about equitable access and the specific patient populations that benefit. Future directions should focus on making these technologies more accessible and affordable to ensure broader adoption across diverse healthcare settings.

The adoption of CAN systems in cervical spine surgeries faces several barriers, yet promising advancements are on the horizon. Current limitations include the need for simultaneous intraoperative fluoroscopic confirmation, cumbersome and costly hardware, and challenges associated with prolonged use, such as physical and visual fatigue. However, technological improvements and the FDA clearance of newer systems, including robotic and AR devices, are poised to overcome these barriers. Manufacturers are developing lighter, more portable, and cost-effective solutions, while innovations like the Cambridge Medical Robotics™ comprehensive packages aim to reduce costs and streamline surgical workflows.^42^ Emerging technologies, such as the Internet of Skills and AI, are expected to enhance remote surgical capabilities and optimize surgical training through VR and AR systems, which have already demonstrated improvements in operative times and performance.

Looking ahead, future CAN systems may incorporate semi-independent, table-mounted robots equipped with multiple arms for various procedures, controlled via AR headsets and sensor gloves that provide enhanced haptic feedback and natural hand motions. These advancements could reduce reliance on fluoroscopic confirmation and improve precision. Innovations such as the robotic simulator developed by Ueda et al in Japan and the Smart Tissue Autonomous Robot (STAR) indicate a future where autonomous surgical systems play a significant role, particularly in complex spinal surgeries.^43,44^ As AR technology continues to evolve, its application in cervical spine surgery will likely expand, offering enhanced pre-operative planning, intraoperative guidance, and educational tools, ultimately improving patient outcomes and OR efficiency. Despite current challenges, the future of CAN technology in spine surgery holds significant potential for advancing surgical precision, safety, and accessibility.

## ORCID iDs

Hania Shahzad https://orcid.org/0000-0002-1643-0828

Shannon Tse https://orcid.org/0000-0003-0370-2377

Sathish Muthu https://orcid.org/0000-0002-7143-4354

Hardeep Singh https://orcid.org/0000-0001-6317-0080

Luca Ambrosio https://orcid.org/0000-0003-2424-1274

Javad Tavakoli https://orcid.org/0000-0002-0696-6530

Sven Yves Vetter https://orcid.org/0000-0001-8024-9276

Philip Louie https://orcid.org/0000-0002-4787-1538

Sangwook Tim Yoon https://orcid.org/0000-0003-1010-6952

Amit Jain https://orcid.org/0000-0002-9983-3365

Hai Le https://orcid.org/0000-0002-9111-9060
